# Vascular stiffness in insulin resistance and obesity

**DOI:** 10.3389/fphys.2015.00231

**Published:** 2015-08-14

**Authors:** Guanghong Jia, Annayya R. Aroor, Vincent G. DeMarco, Luis A. Martinez-Lemus, Gerald A. Meininger, James R. Sowers

**Affiliations:** ^1^Department of Medicine, Division of Endocrinology and Metabolism, University of Missouri School of MedicineColumbia, MO, USA; ^2^Research Service, Harry S Truman Memorial Veterans HospitalColumbia, MO, USA; ^3^Department of Medical Pharmacology and Physiology, University of Missouri School of MedicineColumbia, MO, USA; ^4^Dalton Cardiovascular Research Center, University of MissouriColumbia, MO, USA

**Keywords:** obesity, insulin resistance, vascular resistance, cardiovascular disease

## Abstract

Obesity, insulin resistance, and type 2 diabetes are associated with a substantially increased prevalence of vascular fibrosis and stiffness, with attendant increased risk of cardiovascular and chronic kidney disease. Although the underlying mechanisms and mediators of vascular stiffness are not well understood, accumulating evidence supports the role of metabolic and immune dysregulation related to increased adiposity, activation of the renin angiotensin aldosterone system, reduced bioavailable nitric oxide, increased vascular extracellular matrix (ECM) and ECM remodeling in the pathogenesis of vascular stiffness. This review will give a brief overview of the relationship between obesity, insulin resistance and increased vascular stiffness to provide a contemporary understanding of the proposed underlying mechanisms and potential therapeutic strategies.

## Introduction

Vascular stiffness is a consequence of pathophysiological alterations involving endothelial cells (ECs), vascular smooth muscle cells (VSMCs), extracellular matrix (ECM), and other functional elements of the vessel wall (Brillante et al., 2009; Jia and Sowers, 2014; Villacorta and Chang, 2015). These alterations are believed to occur early and contribute to premature cardiovascular dysfunction and structural alterations leading to increased risk for cardiovascular disease (CVD) morbidity and mortality. The Framingham Heart Study of 2232 participants confirmed vascular stiffness as an independent predictor of CVD morbidity and mortality in the general population, hypertensive patients, the elderly, and patients with end-stage renal disease (Mitchell et al., 2010). Further, increased vascular stiffness is an important early marker for CVD and a predictor of heart attacks and strokes in adults, especially in persons with obesity, insulin resistance and type 2 diabetes mellitus (T2D) (Jia et al., 2014b).

Non-invasive measurements of vascular stiffness usually fall into three categories: (1) analysis of pulse transit time; (2) wave contour of the arterial pulse; and (3) direct measurement of arterial geometry and pressure that corresponds to regional, systemic, and local determination of stiffness (Ray et al., 2014). Recently, the European Society of Hypertension (ESH)/European Society of Cardiology (ESC) guidelines for the management of arterial hypertension suggested the measurement of aortic pulse wave velocity (PWV), which is considered the gold standard method for assessing vascular stiffness, as a tool for assessment of subclinical target organ damage (Mancia et al., 2013). Typical values of PWV in the aorta range from approximately 5 m/s to >15 m/s (Luft, 2012). A fixed threshold value (12 m/s) was proposed as an indication of increased vascular stiffness in the 2007 ESH/ESC hypertension guidelines based on published epidemiological studies (Reference Values for Arterial Stiffness Collaboration, 2010).

Obesity is associated with vascular remodeling and stiffness and has been known to predict increased cardiovascular mortality (Martínez-Martínez et al., 2014). Insulin resistance, a consequence of obesity, has been also shown as an independent risk factor for vascular stiffening and other elements of diabetic vasculopathy (Jia and Sowers, 2014). To this point, the mechanisms and interactions of overweight/obesity and insulin resistance in the regulation of vascular stiffness involve a complex network of interacting factors that are not yet completely understood. Much work needs to be done to clarify initiating and potentiating factors and the progression and vascular distribution of this phenomenon. Therefore, a better understanding of the mechanisms of vascular stiffening in obesity and insulin resistance has great clinical significance. In the present review, we will discuss the roles and mechanisms of vascular stiffness in patients with obesity and insulin resistance to provide a basis for improving understanding of potential therapeutic strategies.

## Obesity and insulin resistance

The overweight/obesity epidemic has led to a marked increase in the incidence of insulin resistance, T2D, and the cardiorenal metabolic syndrome (Leopold, 2013). Overweight is defined as a body mass index (BMI) of 25–29.9, obesity as a BMI > 30, and severe obesity as a BMI > 40 (or ≥35 in the presence of comorbidities) (Forte et al., 2012). Currently, approximately 34.4% of adults and children are overweight in the United States (Forte et al., 2012). Sedentary lifestyle and dietary changes in combination with genetic predisposition are regarded as the major risk factors for obesity (Jia et al., 2014a). A strong association has been observed between obesity and insulin resistance (Herouvi et al., 2013). It has been postulated that with increased abdominal adiposity there is greater lipolytic activity leading to an increase in free fatty acids (FFA), which may inhibit insulin secretion and insulin-stimulated glucose uptake and thus increase the risk for insulin resistance and T2D (Forte et al., 2012; DeMarco et al., 2014). Meanwhile, there is also increased liver synthesis of triglycerides in response to the increased circulating FFA and increased hepatic production of glucose resulting in hyperinsulinemia (Forte et al., 2012; DeMarco et al., 2014). Thus, increased attention to weight control may improve insulin sensitivity and help prevent CVD. However, studies have found an obesity paradox that overweight and obese people have a better prognosis in heart failure, hypertension, end-stage renal disease, and mortality than normal-weight individuals (Morse et al., 2010). For example, approximately 10–25% of obese individuals are metabolically healthy due to preserved insulin sensitivity (Blüher, 2010). These reports suggest that visceral adipose tissue and ectopic fat deposition play an important role in development of insulin resistance in human obesity independently of total body fat mass. Thus, BMI may not be the most accurate index for obesity.

## Vascular stiffness in insulin resistance and obesity

**Figure 1:**
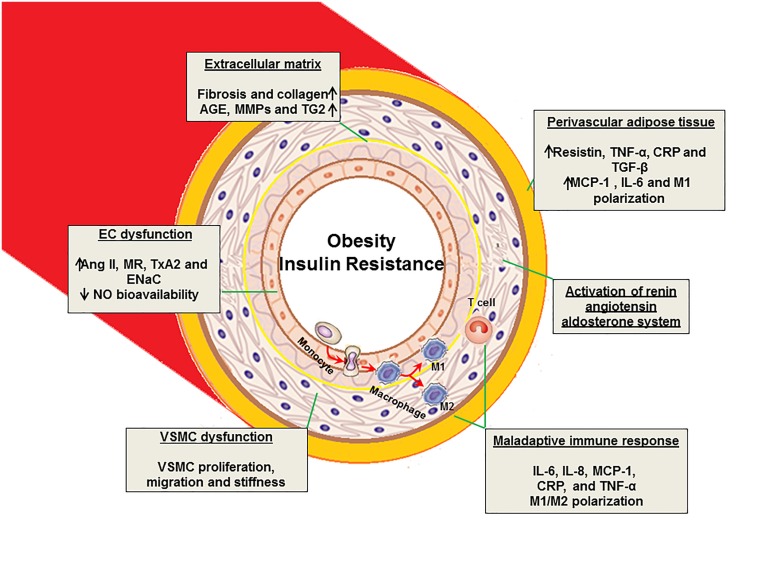
**Proposed mechanisms of vascular stiffness in obesity, insulin resistance, and type 2 diabetes**. EC, endothelial cell; VSMC, vascular smooth muscle cell; AGE, advanced glycation end products; MMPs, matrix metalloproteinase; TG2, tissue transglutaminase; Ang II, angiotensin II; MR, mineralocorticoid receptor; TxA2, thromboxane A2; ENaC, epithelial Na^+^ channel; IL, interleukin; TNF, tumor necrosis factor; NO, nitric oxide; MCP-1, monocyte chemotactic protein-1; CRP, C- reactive protein; TGF-β, transforming growth factor- β.

Obesity and insulin resistance interact and impair vascular function and structure and are linked to endothelial dysfunction, increased artery intima media thickness, and increased vascular stiffness (Herouvi et al., 2013) (Figure 1). It is well accepted that obesity is associated with increased vascular stiffness and associated CVD. Obese individuals exhibit increased vascular stiffness compared with non-obese individuals, and weight loss improves arterial compliance (Villacorta and Chang, 2015). A recent population study showed that skin-fold thickness is a predictor of arterial stiffness in hypertensive patients (Selcuk et al., 2013). Other recent studies implicate dysfunctional perivascular adipose tissue (PVAT) adjacent to the vessel wall in the pathogenesis of vascular stiffness. The PVAT serves not only as a structural component present in most arteries but also as a source of an abundance of molecules with varied paracrine effects (Villacorta and Chang, 2015). In the setting of obesity and insulin resistance, adipocyte hyperplasia is associated with both the infiltration of pro-inflammatory immune cells and a reduced expression of anti-inflammatory factors in the PVAT (Aroor et al., 2013b). The Framingham Offspring and Third Generation cohorts further support the notion that PVAT volume is associated with higher thoracic and abdominal aortic dimensions and increased stiffness even after adjusting for age, sex, and CVD risk factors including BMI and visceral adipose tissue volume (Thanassoulis et al., 2012). These findings support the notion that obesity and PVAT are important contributors to the pathogenesis of vascular stiffness.

Epidemiological studies have demonstrated that hyperinsulinemia or insulin resistance is an independent risk factor for vascular stiffening and other elements of diabetic vasculopathy (Aroor et al., 2013a; Padilla et al., 2015). A report from a cross-sectional study of the relationships between arterial stiffness indexes and serum insulin and glucose tolerance measurements in a biracial population of 4701 men and women aged 45–64 demonstrated these individuals were at increased risk for atherosclerosis. It was also shown that individuals with borderline abnormal glucose intolerance or non-insulin-dependent diabetes mellitus had stiffer arteries than their counterparts with normal glucose tolerance and the decreased elasticity was independent of artery wall thickness (Salomaa et al., 1995). It was suggested that interactive effects of elevated glucose, insulin, and triglycerides may have a combined and synergistic impact on arterial stiffness and play an important role in the early pathophysiology of macro-vascular disease in patients with T2D (Cote et al., 2015). Vascular stiffening in association with obesity and insulin resistance has been observed in all age groups, including children (Tounian et al., 2001; van Popele et al., 2006; Ho et al., 2011). Therefore, obesity-related insulin resistance or T2D is considered a powerful risk factor for occurrence of vascular stiffness and adverse CVD events in both children and adults.

## Vascular stiffness related cardiovascular risk

Data from the Framingham study have established an increased incidence of CVD events with increasing weight in both men and women (Hubert et al., 1983), and these CVD has been strongly associated with insulin resistance and vascular stiffness (DeMarco et al., 2015). However, vascular stiffness independently predicts cardiovascular events (Matsuoka et al., 2005), since increased vascular stiffness is significantly associated with damage to target organs such as the heart, kidney, liver, and brain (Schiffrin, 2004). For example, stiffening of central arteries increases systolic pressure, and decreases diastolic pressure, resulting in increased pulse afterload leading to an increase in left ventricular mass and myocardial oxygen demand. Further, the decrease in diastolic pressure is associated with reduced coronary blood flow during the diastole. These changes have been associated with left ventricular remodeling and fibrosis and thereby cause left ventricular diastolic dysfunction and development of coronary artery disease (Jia et al., 2014b; Bostick et al., 2015). Hypertension is regarded as a major risk factor for vascular remodeling and development of vascular stiffness. Remodeling associated with hypertension has been viewed as an adaptive process involving an increased wall-to-lumen width ratio in response to long-term changes in hemodynamic forces (Weisbrod et al., 2013). However, recent analysis from the Framingham Heart Study and from other studies (Weisbrod et al., 2013; DeMarco et al., 2015) indicated that aortic stiffness precedes hypertension, and progressive structural and functional changes in the aorta may exist prior to the development of increased levels of blood pressure (Liao and Farmer, 2014). Furthermore, initial blood pressure was not independently predictive of subsequent vascular stiffening measured in the same individuals following 4–10 years observation (Kaess et al., 2012). This is an important controversial point in the field and underscores the need to understand the cause and effect relationship between hypertension and aortic stiffness (Liao and Farmer, 2014). Despite this, the evidence suggests that early detection of arterial stiffening certainly helps to identify and reduce risk factors for vascular stiffness and CVD progression.

## Mechanism of vascular stiffness

Obesity, hypertension, dyslipidemia, insulin resistance and activation of renin- angiotensin-aldosterone (RAAS) and sympathetic nervous (SNS) systems are all components of the cardiorenal metabolic syndrome, which is associated with vascular stiffness (Sowers, 2013; Jia and Sowers, 2014; Jia et al., 2014b). Dysregulation of ECs, VSMCs, ECM, and adaptive immune responses in the cardiorenal metabolic syndrome all play a key role in the development of vascular stiffness (Figure 1).

## Adiposity

Excess adipose tissue, in particular abdominal adipose tissue, has been closely linked to the development of vascular stiffness and cardiovascular disease (Strasser et al., 2015). Abnormal production of adipokines participates in the pathogenesis of obesity- and insulin resistance-associated comorbidities, including vascular stiffness and hypertension. Indeed, adipocytes may produce over fifty active substances including monocyte chemotactic protein-1 (MCP-1), tumor necrosis factor-α (TNF-α), interleukin-6 (IL-6), C-reactive protein (CRP), and angiotensin II (Ang II), all of which are released into the circulation and are involved in the regulation of insulin sensitivity, immune responses, vascular function, arterial blood pressure, coagulation and acute inflammation (Zapolski et al., 2011; Jia et al., 2014c) (Figure 1). For example, MCP-1 plays a key role in macrophage infiltration into adipose tissue of obese individuals and is associated with development of insulin resistance (Kanda et al., 2006). Our previous studies demonstrate that consumption of a western diet high in fat and fructose promotes adipose tissue inflammation and results in reduced production of adiponectin, as well as increased secretion of resistin and other inflammatory cytokines, all of which contribute to systemic and cardiac insulin resistance, metabolic cardiomyopathy, and vascular stiffness (Bostick et al., 2014, 2015). Of relevance, resistin, TNF-α, CRP, and IL-6 all inhibit insulin metabolic signaling in these tissues and promote cardiac and vascular stiffness through activation of mitogen activated protein kinase (MAPK), protein kinase C (PKC), rapamycin (mTOR)/S6 kinase 1 (S6K1) and suppressor of cytokine signaling 3 mediated proteasomal degradation of insulin receptor substrate 1 (IRS-1) (Bender et al., 2013).

## EC and VSMC dysfunction

The complex interactions between EC and VSMC are important for the modulation of vascular function and tone. For example, EC production of nitric oxide (NO) has a vasodilatory effect and anti-atherogenic properties, including inhibition of VSMC proliferation and migration, platelet activation and adhesion and leukocyte adhesion and migration (Herouvi et al., 2013). To this point, NO diffuses into neighboring VSMCs, activating guanylyl cyclase to produce cyclic guanosine monophosphate (cGMP) and activate kinases responsible for vascular relaxation (Bender et al., 2013). Indeed, vascular tone is tightly controlled by EC secretion of vasodilatory substances, such as NO, endothelium-derived hyperpolarizing factor (EDHF), prostacyclin (PGI_2_), and vasoconstrictor substances, such as angiotensin II (Ang II), and thromboxane A2 (Creager et al., 2003; Mudau et al., 2012). Further, EC dysfunction and increased stiffness (Fels et al., 2014; Oberleithner, 2014) have been proposed to mediate the changes in the vasculature that lead to fibrosis and stiffness in obesity, insulin resistance and T2D.

VSMCs are the predominant cell type found in the medial layer of the vessel wall and are a target of insulin metabolic and growth signaling (Doronzo et al., 2004; Jia and Sowers, 2014), as well as the target of most vascular therapies aimed at reducing mean arterial pressure. Insulin normally induces vasodilation in VSMCs through insulin metabolic signaling that includes IRS-1/phosphatidylinositide 3-kinases (PI3K), protein kinase B (Akt) and cGMP signaling pathways. This signaling leads to a reduction of free intracellular calcium and reduction in contractile apparatus calcium sensitivity (Doronzo et al., 2004). Thus, insulin resistance in VSMCs impairs vascular vasodilation. Indeed, studies from insulin resistant obese Zucker rats have shown that VSMCs from these rats manifested greater concentrations of reactive oxygen species and have impaired activation of the NO/cGMP/PKG pathway when compared to VSMC from insulin-sensitive Zucker Lean rats (Doronzo et al., 2004). Our recent data also showed that a western diet high in fat and refined carbohydrates impaired mouse aortic endothelium-dependent and endothelium-independent vasodilation by protein kinase B/endothelial NO synthase (eNOS) signaling pathways (DeMarco et al., 2015). These observations provide a biochemical basis by which obesity and insulin resistance in vascular cells could lead to development of vascular stiffening.

## ECM

Alterations in ECM composition and structure are important contributors to vascular compliance and vascular stiffness. Transforming growth factor (TGF-β) and connective tissue growth factor (CTGF) are well-known profibrotic factors that can stimulate synthesis of ECM proteins, such as fibronectin and collagens, under conditions of obesity, insulin resistance, and T2D (Jia et al., 2015). Studies have also found that advanced glycosylation end-products (AGEs) enhance collagen content and cross linking, and induce changes in mechanical properties of the ECM (Jia et al., 2014b). Indeed, AGEs promote a decrease in the rate of ECM degradation, an increase in the production of nascent ECM and an increase in cross-linking of the extracellular proteins (Torjesen et al., 2014). In the context of vascular stiffening, the matrix metalloproteinases (MMPs) are involved in the regulation of the structural integrity of the ECM. The MMPs are a family of enzymes that proteolytically degrade ECM and participate in this remodeling of vessel wall (DeMarco et al., 2015). The increase in MMPs in vessels from insulin resistant and diabetic animals has been reported to be accompanied by pronounced generation of angiostatin, and the reduction of microvascular density was associated with impaired vasorelaxation (Chung et al., 2009; Paik et al., 2013; Wang et al., 2014). In addition, tissue transglutaminase (TG2) plays a key role in promoting vascular stiffness by increasing cell surface and ECM cross linking activity in the vasculature (Deenadayalu et al., 2012). Thus, deleterious remodeling of ECM results from numerous maladaptive processes that are not limited purely to changes in content of a particular ECM protein type.

## RAAS

It has been recognized that inappropriate neurohumoral activation including both Ang II and aldosterone promote vascular stiffness in obesity and T2D. Both hormones directly modulate vascular stiffness by upregulation of nicotinamide adenine dinucleotide phosphate (NADPH) oxidase activity and reduction of NO bioavailability, thereby promoting oxidative stress and vascular dysfunction (Manrique et al., 2013). Recently, it was shown that prolonged exposure to increased mitochondrial oxidative stress decreased aortic compliance and induced cardiac dysfunction (Zhou et al., 2012). Specifically, the data elucidated the significance of lifelong superoxide dismutase 2 deficiency on the phenotype, function, and molecular signaling pathways of aortic SMCs. These results further showed how oxidative stress promotes aortic stiffening by inducing vascular wall remodeling, intrinsic changes in SMC stiffness, and aortic SMC apoptosis (Zhou et al., 2012). Aldosterone and increased salt in the diet also increase epithelial Na^+^ channel (ENaC) expression on the EC surface leading to reduced NO production that is associated with increases in cortical stiffness of the cytoskeleton (Kusche-Vihrog et al., 2014) (Figure 1). Some of our studies have explored the signaling pathways by which enhanced tissue RAAS contributed to insulin resistance and cardiovascular stiffness. For example, Ang II increases serine phosphorylation of IRS-1 and inhibits the insulin-stimulated phosphorylation of eNOS through activation of S6K1 signaling pathway. Also, an inhibitor of mTOR (rapamycin) attenuates the Ang II-stimulated phosphorylation of p70S6K and IRS-1 and blocks the ability of Ang II to impair insulin-stimulated phosphorylation of eNOS and NO dependent-arteriole vasodilation (Kim et al., 2012). Thus, we conclude that activation of mTOR/p70S6K by Ang II and aldosterone in vascular ECs and VSMCs may contribute to the impairment of insulin-stimulated vasodilation through phosphorylation of IRS-1 (Cote et al., 2013). Indeed, adipose tissue expresses all components of RAAS, such as renin, angiotensin converting enzyme 1, angiotensin converting enzyme 2, and angiotensin receptor (AT) 1, AT2, as well as the Mas receptor (Grobe et al., 2013). Global knockout (KO) of AT1 resulted in the attenuation of weight gain and adipose deposition during high-fat feeding. Conversely, AT2 KO had no obvious effect on adipose deposition (Grobe et al., 2013). Furthermore, Mas receptor KO, which is the receptor Ang-(1–7), has a complex effect on body composition, including an increase in abdominal fat mass, and decreased glucose tolerance and insulin sensitivity (Santos et al., 2008). Thus, further studies are necessary to understand RAAS in the pathogenesis of vascular stiffness.

## Maladaptive immune responses

Vascular stiffness is associated with chronic inflammatory disease in the vessel wall characterized by an activation of both the innate and adaptive immune systems, which are composed of diverse cellular components, including granulocytes, mast cells, monocytes, macrophages, and natural killer cells (Aroor et al., 2013b) (Figure 1). For example, activated T cells can be sub-typed according to their cytokine profile. T helper (Th) 1 cells secrete IL-2, TNF-β, and IFN-γ, whereas Th2 cells typically produce IL-4, -5, -6, and -10 (Ait-Oufella et al., 2006). Increased Th cell secretion of cytokines, chemokines, and growth factors leads to an inflammatory process that may lead to fragmentation of elastic membranes and destruction of cell-protective matrix layers. However, CD4^+^CD25^+^Foxp3^+^ regulatory T cells (Tregs) can protect the vascular cells by immunosuppression, cell contact–dependent suppression, and functional modification or killing of activated protein C (He et al., 2010). Furthermore, macrophage polarization toward an enhanced M1 pro-inflammatory response and suppression of an M2 anti-inflammatory response occurs in insulin resistance and obesity (Aroor et al., 2013a,b). The pro-inflammatory M1 macrophages secrete inflammatory cytokines such as TNF-α that cause insulin resistance. In contrast, M2 macrophages secrete IL-10, which can improve the insulin signaling impaired by pro-inflammatory cytokines (Aroor et al., 2013a,b). It has been shown that an acute high fat diet boosts growth and promotes hematopoietic expansion and differentiation in fetal mice, including lymphoid cells (CD3þ/B220þ) and myeloid cells (Gr1þ/Ter119þ) (Kamimae-Lanning et al., 2015). However, obesity diminishes endothelial progenitor cells level, impaired the recovery of damaged endothelium, and suppressed endothelial progenitor cell angiogenesis ability, resulting in left ventricular remodeling and cardiac dysfunction (Tsai et al., 2012). The link between adaptive immune responses, hematopoietic abnormality, and vascular stiffness offers possibilities for identification of the origins of vascular dysfunction and altered vascular stiffness and may allow for development of novel targeted therapeutic interventions.

## Gender, vascular stiffness, insulin resistance, and obesity

Non-diabetic premenopausal women exhibit lower incidence of cardiovascular complications compared to age matched men. However, this protection is lost under the setting of obesity and T2D (Manrique et al., 2013; DeMarco et al., 2015). Indeed, women display increased risk for diastolic dysfunction than men in the setting of obesity and T2D. Moreover, higher ventricular stiffness seen in obese women may contribute to increased incidence of diastolic dysfunction (DeMarco et al., 2015; Jia et al., 2015). Further, left ventricular mass correlates positively with insulin resistance and glucose intolerance, especially in women (Manrique et al., 2013). Therefore, significance of studies focused on sex differences in cardiovascular dysfunction is increasingly recognized (Clayton and Collins, 2014).

Although the mechanisms that contribute to vascular stiffness in obese premenopausal women are poorly understood, upregulation of RAAS signaling may be important in this regard (Aroor et al., 2013a). Aldosterone levels were found to be higher in women and to be positively associated with cardiac structural remodeling in females, but not in males (Vasan et al., 2004). Aldosterone promotes cardiac and vascular stiffness and administration of the aldosterone antagonist spironolactone prevents both cardiac and vascular stiffness in a clinically translational model of western diet-induced obesity in female mice (Bostick et al., 2015; DeMarco et al., 2015). The improvement in vascular stiffness is also accompanied by suppression of M1 macrophage polarization, decreased vascular oxidative stress and improvement in insulin metabolic signaling (DeMarco et al., 2015).

## Conclusion

Vascular stiffness is increased in conditions of insulin resistance and obesity and independently increases the risk of developing hypertension, coronary heart disease, and CVD morbidity and mortality. The underlying pathophysiology of vascular stiffness in insulin resistance and obesity involves activation of RAAS, adipocyte inflammation, abnormalities in vascular cells, ECM and maladaptive immune responses, all of which increase the risk of vascular stiffness associated CVD events (Figure 1). Future therapeutic strategies should emphasize the need to control glycemia, as well as adopt healthier lifestyles incorporating better nutrition, weight control, more physical activity and cigarette smoking cessation. A better understanding of the underlying mechanisms leading to vascular stiffness and associated cardiovascular diseases may disclose new strategies to controlling the morbidity and mortality in these patients.

## Conflict of interest statement

The authors declare that the research was conducted in the absence of any commercial or financial relationships that could be construed as a potential conflict of interest.
